# Hydrogen Peroxide and Redox Regulation of Developments

**DOI:** 10.3390/antiox7110159

**Published:** 2018-11-06

**Authors:** Christine Rampon, Michel Volovitch, Alain Joliot, Sophie Vriz

**Affiliations:** 1 Center for Interdisciplinary Research in Biology (CIRB), College de France, CNRS, INSERM, PSL Research University, 75231 Paris, France; Christine.rampon@college-de-france.fr (C.R.); Michel.volovitch@ens.fr (M.V.); alain.joliot@college-de-france.fr (A.J.); 2 Sorbonne Paris Cité, Univ Paris Diderot, Biology Department, 75205 Paris CEDEX 13, France; 3 École Normale Supérieure, Department of Biology, PSL Research University, 75005 Paris, France

**Keywords:** H_2_O_2_, redox signalling, development, regeneration, adult stem cells, metazoan

## Abstract

Reactive oxygen species (ROS), which were originally classified as exclusively deleterious compounds, have gained increasing interest in the recent years given their action as *bona fide* signalling molecules. The main target of ROS action is the reversible oxidation of cysteines, leading to the formation of disulfide bonds, which modulate protein conformation and activity. ROS, endowed with signalling properties, are mainly produced by NADPH oxidases (NOXs) at the plasma membrane, but their action also involves a complex machinery of multiple redox-sensitive protein families that differ in their subcellular localization and their activity. Given that the levels and distribution of ROS are highly dynamic, in part due to their limited stability, the development of various fluorescent ROS sensors, some of which are quantitative (ratiometric), represents a clear breakthrough in the field and have been adapted to both ex vivo and in vivo applications. The physiological implication of ROS signalling will be presented mainly in the frame of morphogenetic processes, embryogenesis, regeneration, and stem cell differentiation. Gain and loss of function, as well as pharmacological strategies, have demonstrated the wide but specific requirement of ROS signalling at multiple stages of these processes and its intricate relationship with other well-known signalling pathways.

## 1. Introduction

For a long time, reactive oxygen species (ROS), including hydrogen peroxide (H_2_O_2_), were considered deleterious molecules. Emphasis was given to their role in neutrophils where they are produced to contribute to anti-microbial defence [1], and extensive studies have been performed on ROS over-production due to mitochondrial dysfunction in neurological disorders or cancer progression [2,3,4]. Consistent with these detrimental functions, attention has been almost exclusively focused on their toxicity, and many studies strengthened this aspect of redox biology. However, pioneer works highlighted a new role of ROS in signalling, which led to the emergence of the redox signalling field [5,6]; recent reviews in [7,8]. Redox signalling soon also proved to be important during animal development for review [9,10]. In 2017, Helmut Sies, a pioneer in redox biology, reviewed the topic and developed the concept of oxidative eustress (physiological redox signalling) and oxidative distress (pathophysiological disrupted redox signalling), bringing the two faces of ROS back together [11]. As recently noted [12], a new reading of the past literature might shed a new light on the tenets of redox signalling. Relevant issues are the nature of the ROS invoked, the accurate localization of its site of production, and its concentration, spreading and dynamics in the context of a defined physiological process. The present review focuses on H_2_O_2_, a central ROS in redox signalling during development and regeneration in metazoans, and its interplay with the redox machinery. We will not address the role of other reactive species, and readers are referred to excellent reviews on Reactive Nitrogen Species (RNS) or oxidized lipids recently published [13,14].

H_2_O_2_ is the major ROS produced by cells that acts in signalling pathways as a second messenger [11,15,16,17]. H_2_O_2_ is a by-product of many oxidative reactions, such as oxidative protein folding in the endoplasmic reticulum (ER) and peroxisomal enzyme activities. For signalling purposes, the main sources of H_2_O_2_ are the mitochondrial respiratory chain and NADPH oxidases (NOXs) [18]. NOXs are trans-membrane proteins that use cytosolic NADPH as an electron donor. NOXs belong to multi-component complexes that generate either O_2_^−^ (NOX 1, 2, 3 and 5) or H_2_O_2_ (NOX 4, DUOX 1 and 2) upon appropriate stimulation (by growth factors, cytokines…) [19,20]. Even when the primary product of NOX activity is O_2_^−^, it is largely and immediately transformed into H_2_O_2_ by a superoxide dismutase (SOD) enzyme physically associated with NOX, or it dismutates spontaneously at low pH levels. Several NOXs are located at the plasma membrane, which is a hub for cell signalling. In this case, H_2_O_2_ is delivered in the extracellular space, a somehow puzzling situation considering that most known H_2_O_2_ targets localize in the cell interior. It was first thought that H_2_O_2_ could pass from the extracellular to the intracellular milieu by passive diffusion through the plasma membrane, but it was later shown that H_2_O_2_ has poor lipid membrane diffusion capacities and crosses into cells via aquaporin channels [21,22,23]. This facilitated transport of H_2_O_2_ across the plasma membrane is itself subject to redox regulation [24], and further investigations are needed to better understand the role of aquaporins in redox signalling. The unique and specific enzyme for H_2_O_2_ degradation into H_2_O is the ubiquitously expressed protein catalase. It mainly localizes in the peroxisome where it is devoted to the reduction of excess H_2_O_2_ produced there. However, it can also be secreted by an unknown mechanism and associate with the plasma membrane [25,26,27] or spread in the extracellular milieu [28].

The main physiological target of H_2_O_2_ action is the reversible oxidation of cysteine residues in proteins. Modification only occurs on the thiolate anion form (S^−^). However, at physiological pH, most cysteines are protonated and thus react weakly with H_2_O_2_. However, the pK_a_ of cysteine greatly depends on its protein environment and can reach several units below ~8.5, the approximate value of cysteine alone [29], making these residues ionized and reactive. H_2_O_2_ oxidizes the thiolate anion to produce sulfenic acid, which is highly reactive and readily forms a disulfide bond in contact with accessible –SH group. Reciprocally, in reducing conditions, disulfide bonds can be easily cleaved to restore the thiol functions. As oxidative condition increases, sulfenic acid will further oxidize to sulfinic and ultimately sulfonic derivatives. These two reactions are generally irreversible deleterious modifications; however, exceptions were reported for sulfinic derivatives (see below). Redox signalling depends both on the local concentration of H_2_O_2_ and the state (protonated or deprotonated) of the cysteine. Although some cysteines can be directly oxidized by H_2_O_2,_ most of them require prior activation to be deprotonated, involving additional redox-sensitive relays. The best candidates for this relay function appear to be proteins first identified as antioxidant safe-guarders [30,31,32,33,34,35,36] reviews in [37,38,39,40,41], and they will be discussed below. It is now clear that the role of H_2_O_2_ signalling in oxidative eustress has to integrate the entire redox machine.

## 2. The Redox Machine

**Figure 1:**
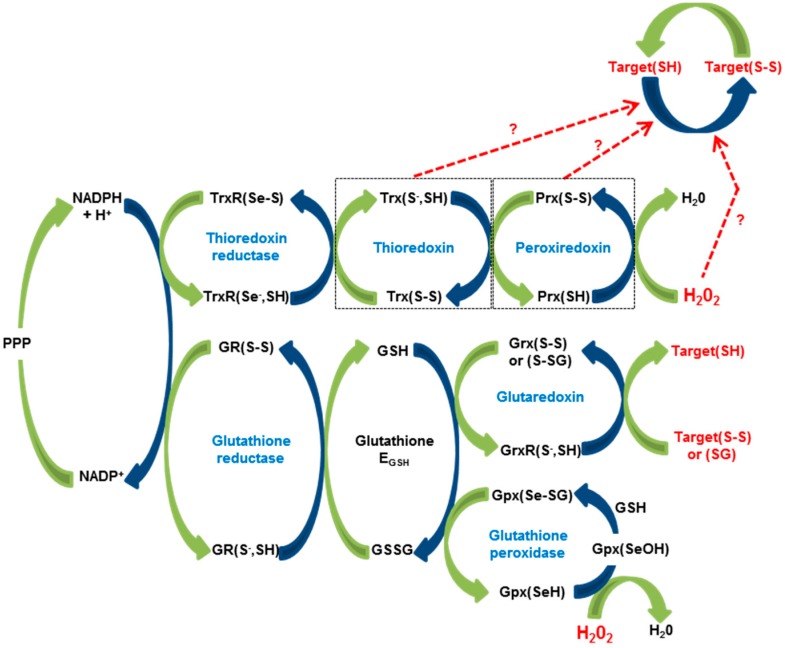
The redox machinery. Interconnection of redox couples from H_2_O_2_ to thiol targets are represented. H_2_O_2_ is a by-product of oxidative reactions. Major sources include mitochondrial respiratory chain and NOXs for review [18]. PPP: Pentose Phosphate Pathway.

The central redox machine contains at least six main protein families: thioredoxin reductases (TrxRs), thioredoxins (Trxs), peroxiredoxins (Prxs), glutathione reductases (GRs), glutaredoxins (Grxs) and glutathione peroxidases (Gpxs) (Figure 1) [for general reviews, see [42,43,44,45]. Moreover, as schematized in Figure 1, the activities of all enzymes in the redox machine are interconnected (some additional branches between cycles have been omitted), and the final outcome of thiol-oxidation reactions depends on many parameters, making computational modelling useful but hampering genetic approaches.

As mentioned in the introduction, the central redox machine has pleiotropic functions. In addition to detoxification of harmful amounts of ROS, they also act as sensors of oxidant concentrations and can even acquire new functions, such as chaperones activity for some Prxs [46] for a review. This sensor and/or transducer functions are very important given that the vast majority of redox-sensitive proteins are poorly sensitive to direct oxidation by H_2_O_2_ (a possible exception, PTP1B, is discussed in [39]). Prxs have attracted considerable attention as potent mediators redox signals, as first established in yeast [31,47,48,49,50], and some years after in mammals. Ledgerwood and colleagues demonstrated that Prx1 participates in the propagation of peroxide signals via disulfide exchange with the target kinase ASK1 [51], and the group of Tobias Dick showed that Prx2 forms a redox relay for H_2_O_2_ signalling together with the transcription factor STAT3 [36]. Very recently, the same group demonstrated that the relay activity of cytosolic Prxs (1 and 2) is not dependent on Trx1 or TrxR1 but is based on transient disulfide conjugates with protein targets and occurs mainly in conditions of fast response to small variations in H_2_O_2_ [52].

## 3. Seeing Is Believing

A critical step to model redox signalling is to determine the spatiotemporal localization and amount of the different protagonists. Several synthetic dyes were actively used to measure ROS and RNS [53,54,55]. However, these dyes are often poorly specific, do not penetrate in tissue, or are unstable. Moreover, their reaction with ROS/RNS is irreversible. In the last decade, a major effort was devoted to develop genetically encoded fluorescent biosensors for the redox machine elements.

### 3.1. H_2_O_2_ Sensors

For all ROS, ex vivo and in vivo measurements of H_2_O_2_ concentration are challenging due to its short half-life, fast-spreading and high reactivity. The development of a genetically encoded fluorescent biosensor specific for H_2_O_2_ revolutionized the field. It provides access to the dynamics of H_2_O_2_ concentration in living systems and its modulation by genetic or chemical approaches. This goal was first achieved by Vsevolod Belousov who designed the HyPer probe [56]. The HyPer biosensor is based on the fusion of a circularly permutated fluorescent protein (cpYFP) with the H_2_O_2_-sensing domain of *E. coli* OxyR. Two cysteines of OxyR moiety form a disulfide bond in the presence of H_2_O_2_ and the resulting conformational change induces a modification of cpYFP spectra, which allows a ratiometric measurement of H_2_O_2_ levels. Advantages of this probe are its high sensitivity (nanomolar), its reversibility and its fast reaction rate constant. Moreover, ratiometric measurement is independent of the expression level. The main drawback of this sensor is its sensitivity to pH. To circumvent this problem, a cysteine-mutated form of HyPer (SypHer), which is still sensitive to pH but no longer to H_2_O_2_, can be used as a control or to measure pH in vivo [57]. Since the initial version, HyPer probe has evolved, and the HyPer family currently includes members with different spectral and redox properties [58].

**Figure 2:**
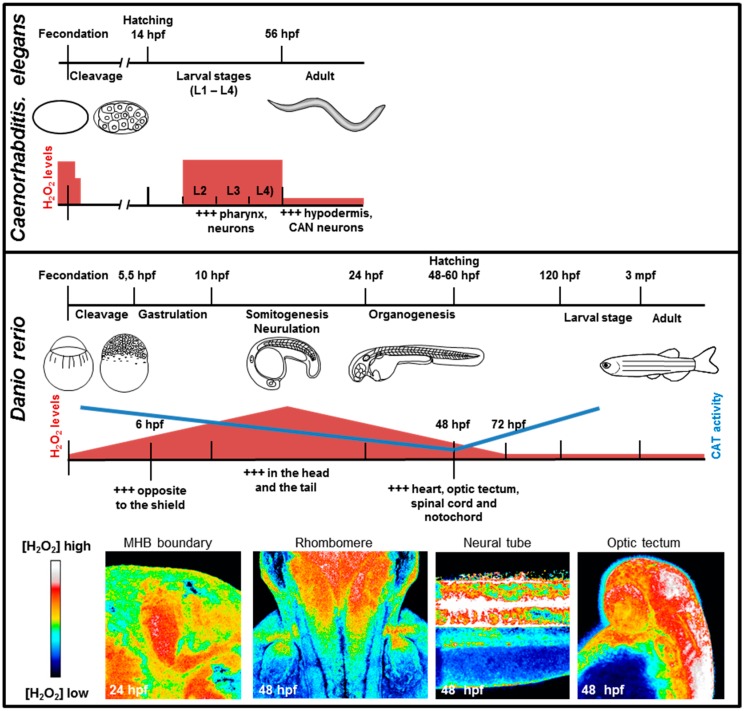
H_2_O_2_ detection during development. Upper panel: H_2_O_2_ detection during *C. elegans* development. Adapted from [62] and [60]. Middle panel: H_2_O_2_ levels and catalase activity during *Danio rerio* development. Adapted from [61]. Lower panel: HyPer fish reveal spatio-temporal dynamic and gradients of H_2_O_2_ during neural development. hpf: hours post fertilization, mpf: month post fertilization.

When expressed in *Xenopus laevis* oocytes, HyPer revealed an oscillating production of H_2_O_2_ induced by fertilization. This production of H_2_O_2_ is of mitochondrial origin, dependent on calcium waves initiated by fertilization and involved in cell cycle progression at the beginning of development [59]. HyPer was also expressed by transgenesis in two animal models (nematode and fish), where it revealed a highly dynamic fluctuation in H_2_O_2_ levels during embryonic and post-embryonic development. In *Caenorhabditis elegans* (where HyPer expression was under the control of the ubiquitous RPL-21 promoter), H_2_O_2_ levels were high during larval development (in the head, notably in the pharynx and neurons), strongly decreased at the transition to the adult stage, and remained low during most of the reproductive period [60]. A similar pattern was observed in *Danio rerio* transgenic animals with high levels of H_2_O_2_ during development and a massive reduction at 3 days post fertilization (dpf) when most of the developmental programmes have ended. Notably, in fish and nematode, HyPer revealed a highly dynamic pattern of H_2_O_2_ levels in the developing nervous system [61] (Figure 2).

Another type of H_2_O_2_ sensor was developed from a fusion between roGFP2 (a redox-sensitive GFP) and Orp1, the yeast H_2_O_2_ sensor and modulator of redox-sensitive transcription factor Yap1 [34]. Orp1 is sensitive to H_2_O_2_; once oxidized, Orp1 promotes the nearby oxidation of roGFP2 (as it does for Yap1), resulting in a shift of roGFP2 spectral properties. Compared with HyPer, this biosensor is insensitive to pH but less sensitive to H_2_O_2_. This lower affinity for H_2_O_2_ was overcome by fusion of roGFP2 to the yeast Prx Tsa2 (Tsa2ΔC_R_) [63]. roGFP2-Orp1 has been successfully used to measure H_2_O_2_ in developing and adult *Drosophila* [64]. One of the advantages of genetically encoded sensors is their ability to be addressed to a cell-specific compartment upon fusion with appropriate targeting sequences. Differential targeting into either the cytosol or the mitochondria allowed Albrecht et al. to demonstrate the heterogeneity of H_2_O_2_ levels depending on the tissue and that H_2_O_2_ level is not coupled with the redox state of glutathione during development [64]. Cytosol/mitochondria expression of roGFP2-Orp1 in the germline of *Caenorhabditis elegans* revealed an increase in H_2_O_2_ levels in the proximal side of the germline and a peak within the oocytes and in the zygote [65]. An elegant approach preserving the redox status of roGFP2-Orp1 during tissue cryo-section allowed H_2_O_2_ measurements in mammalian development and adult tissues [66]. This strategy is very promising to acquire redox maps of non-optically accessible tissue. roGFP2-Orp1 was also targeted to zebrafish cardiomyocytes in different compartments (nucleus, mitochondria and cytosol) to follow H_2_O_2_ level variations during cardiac function and upon pharmaceutical treatments, demonstrating the interest of this H_2_O_2_ probe to score oxidant or antioxidant molecules [67].

### 3.2. Glutathione Redox Potential Sensors

Glutathione plays a key role in cellular thiol-disulfide exchange reactions, and the GSH/GSSG ratio is considered a good indicator of redox balance (E_GSH_) (Figure 1). A fluorescent sensor for E_GSH_ was generated by fusion of the roGFP2 with the human glutaredoxin-1 (Grx1). roGFP2 alone exhibits a slow response to redox changes. Grx1 fusion to roGFP2 resulted in a rapid equilibrium between the GSH/GSSG couple and the reporting redox couple (roGFP2red and roGFP2ox), thus reflecting the level of E_GSH_ [68]. This sensor was introduced in several species. In *Drosophila*, it was addressed to mitochondria and cytosol to compare E_GSH_ with H_2_O_2_ levels in developing structures and adults [64]. Live imaging of the third-instar larvae revealed high variations in mitochondrial E_GSH_ amongst different tissues, whereas the cytosolic E_GSH_ was almost constant [64]. Transgenic *C. elegans* expressing a cytosolic form of Grx1-roGFP2 under a ubiquitous promoter was used to analyse E_GSH_ during development [62]. E_GSH_ decreases globally during development and then remains constant in adult except in the spermathecae where fertilization occurs [62].

### 3.3. NADPH Sensor: iNap

NADPH is a key element in the redox machine as a final electron donor for thiol oxidation by H_2_O_2_. A genetically encoded fluorescent indicator for NADPH (iNap sensor) was developed by mutagenesis of the ligand binding site of the NADH/NAD+ sensor SoNar to switch the selectivity from NADH to NADPH [69]. iNap can be used in vivo, and a proof of concept experiment was performed in a wound healing assay in zebrafish larvae. In combination with a red version of HyPer, the iNap biosensor revealed the concomitant decrease of NADPH levels after tissue wounding with an increase in H_2_O_2_ levels, which is consistent with NADPH consumption by NOXs during wound healing [69].

All these sensors provide invaluable information on cellular redox status in vivo. However, it is worth reminding that all H_2_O_2_ sensors consume H_2_O_2_ to measure its concentration. Thus, there is room to improve these systems in particular through increased sensitivity. Only one molecule of H_2_O_2_ is consumed to modify each molecule of sensor for both HyPer and roGFP2-Orp1. However, for the later, it also depends on the local redox potential. This is a clear illustration of a common difficulty: seeing is modifying.

## 4. Redox Signalling in Animal Development and Regeneration

H_2_O_2_ is generated in response to many stimuli, including cytokine or growth factors, which are involved in embryonic development and adult homeostasis. Given redundancy in the redox machinery components, most single Knock out (KOs) in mice are viable, and embryonic development generally occurs normally [70]. Metazoan development can be divided into 3 phases: (1) fertilization and cleavage period, (2) gastrulation, and (3) morphogenesis. At the adult stage, most tissues are continuously renewed and, in some species, rebuilt after amputation. This section will describe the state of the art for the role of redox signalling during these processes. Many molecular targets of redox signalling are now known, as well as the mechanisms by which their redox balance influences the pathways they belong to, for which excellent recent reviews exist [7,8,9,10,71]. The role of RNS and oxidative stress in pathologies of the nervous system, which are not discussed here, are extensively reviewed in [72,73,74]. Finally, it is worth mentioning that some developmental effects of redox signalling in the brain only become apparent much later (as for instance in the case of critical periods [75], mental illnesses, such as schizophrenia [76], or autism-like behaviours in mouse [77]). These effects have only been analysed in the context of their dysregulation, but their normal progress certainly warrants better examination.

### 4.1. Embryonic Development

#### 4.1.1. NADPH Oxidase Complexes in Embryonic Development

Given the importance of NOXs in anti-microbial defence, inflammation, disorders including cancers, and more generally in the maintenance of redox balance, most studies on these enzymes in metazoans focus on adult expression. Some NOXs exhibit broad distribution among tissues (Nox2 was first believed to be exclusively expressed in neutrophils and macrophages and is currently known to present the largest distribution). Others are more restricted (Nox3 is predominantly expressed in the inner ear), but none of them is ubiquitously expressed. Constitutive expression coexists with induction phenomena (for review see [19,78]. NOX expression also greatly varies during development (Table 1). The most detailed study was performed by qPCR and in situ hybridization in developing zebrafish [79]. Unlike the uniform and homogeneous expression of Nox2, Nox1 and Nox5 expression is high during gastrulation and then decreases to a basal level upon morphogenesis. During this period of development, Nox1 expression is increased in the brain. The expression of dual oxidase (Duox), which is a member of the NOX family, is increased during late morphogenesis [79]. In rodents, Duox expression patterns were described in embryonic thyroid [80] and Nox2 and 4 were looked at during limb formation [81]. Though useful, these expression data did not provide a clear picture of the physiological function of these enzymes. Till the advent of tools enabling to study redox biology in live organisms (see below), the contribution of H_2_O_2_ signalling to developmental processes was essentially analysed in embryonic stem (ES) cells. More than 10 years ago, it was proven that ES cell differentiation into cardiac lineage was dependent on NOX enzymes [82,83]. More examples of ES cell sensitivity to redox potential are now known, which can be found in recent reviews on the subject [84,85].

The use of the HyPer probe in a live animal revealed a surprisingly high level of oxidation during zebrafish development, and H_2_O_2_ levels proved to be heterogeneous and dynamic in space and time [61] (Figure 2). H_2_O_2_ levels occasionally exhibit a graded distribution with clear functional outcome. This distribution occurs in the embryonic tectum where the organization of the retinotectal projections is impaired by Pan NOX inhibition [61]. Nox2 invalidation using the CRISPR/Cas9 strategy induced the same phenotype [103], confirming Nox2 involvement in axon pathfinding during zebrafish development. Very recently, the group of E. Amaya proved that Duox activity was necessary for the development of zebrafish thyroid [104], and that ROS play a role in Xenopus mesoderm formation [59]. A role for Nox enzymes was also evidenced ex vivo for the differentiation of chondrocytes [81] or endometrial cells [105], the establishment of rat hippocampal neuron polarity in culture [106,107], the in vitro maturation of rat cerebellar granule neurons [108], and growth cone dynamics in *Aplysia* neuron [109,110,111]. Nox were also shown to be involved in epithelial-to-mesenchymal transition in normal or tumoral epithelial cell lines [112,113].

#### 4.1.2. Catalase, Superoxide Dismutases and Glutathione Systems in Embryonic Development

Catalase, Sod1 and GPx expression and activities have been analysed during mice development from 8 days of gestation to adulthood [114]. mRNAs of these 3 proteins increase during somitogenesis. Then, catalase activity increases after birth, whereas Sod1 and GPx activities reach a plateau. In zebrafish, SOD activity is globally constant throughout morphogenesis until 7 dpf, whereas an increase in catalase activity is observed from 48 hpf onward when morphogenesis is almost completed [61], (Figure 2). In *Drosophila*, catalase protein is minimally detectable during embryogenesis and enhanced during the third instar larval stage and after the first day of pupal development [115]. It thus appears that low levels of catalase expression during development and high levels in mature tissues represent a general property that has been verified in mice, *Drosophila* and zebrafish.

GSH/GSSG balance is a crucial redox parameter during development [for review: [116]. Embryos from mutant mice invalidated for the enzymes responsible for GSH synthesis fail to gastrulate, do not form mesoderm, develop distal apoptosis, and die before day 8.5 [117,118]. In zebrafish, the total amount of glutathione doubles during embryonic development. The redox potential (E_GSH_) is high in eggs and late larval stage but very low during morphogenesis [119]. This study defined 4 GSH contexts during embryonic development: (1) high E_GSH_ and low GSH in mature oocytes, (2) low E_GSH_ and low GSH from mid-blastula to 24 hpf, (3) high E_GSH_ and high GSH during organogenesis (30–48 hpf) and (4) high E_GSH_ and high GSH in mature larvae.

Grx patterns of expression during development have not been systematically investigated, but expression of Grx2 and its isoforms in vertebrate tissues at various stages (mostly adults) is described in [120,121,122,123]. Their role during development has been demonstrated in different models. One of the first hint (though outside metazoan) was that Grx1 affects cell fate decision in the culmination process in *Dictyostelium discoideum* [124]. It was subsequently shown that knocking down (KD) cytosolic Grx2 in zebrafish embryo impaired the development of the central nervous system. Grx2 is indeed essential for neuronal differentiation, survival and axon growth [87]. In mammals, Grx2 controls axon growth via a dithiol-disulfide switch affecting the conformation of CRMP2, a mediator of semaphorin-plexin signalling pathway [125]. The same Grx2 isoform is also required for correct wiring of embryonic vasculature in zebrafish by de-glutathionylation of the active site of the NAD-dependent deacetylase Sirtuin-1 [126]. Grx2 is also implicated in embryonic heart formation. Grx2 KD in zebrafish embryo prevented neural crest cell migration into the primary heart field, impairing heart looping [127]. The role of other members of the Grx family was also described in erythropoiesis. Grx5 is essential to zebrafish haeme synthesis through assembly of the Fe-S cluster, and this role is apparently conserved in humans [89]. Grx3 is also crucial to red blood cell formation in zebrafish as demonstrated by the reduced number of erythrocytes in embryos treated with Grx3 morpholinos and is required in human cells for the biogenesis of Fe-S proteins, as demonstrated by silencing Grx3 expression in HeLa cells [88]. Grx3 KO in mice induces a delay in development and eventually death approximately 12.5 days of gestation. Ex vivo analysis of Grx3^−/−^ cells reveals impaired growth and cell cycle progression at the G2/M transition [128]. The loss of Grx3 also disturbs the development of mammary alveoli during pregnancy and lactation [129]. In addition to these roles during development proper, Grx proteins are also involved in various physio-pathological processes (for review [130]).

#### 4.1.3. Thioredoxin System in Embryonic Development

Two distinct thioredoxin/thioredoxin reductase systems, Trx1 and Trx2, are present in mammalian cells in the cytosol and mitochondria. Trx1 and Trx2 are ubiquitously expressed, but Trx2 is expressed at higher levels in metabolically active tissues, such as heart, brain, and liver [131]. Interestingly, in mice, Trx1 (the cytosolic and nuclear Trx) KO is embryonic lethal early after implantation [132] and presents a dramatically reduced proliferation of inner mass cells. Trx2 (the mitochondrial form) KO is also embryonic lethal. Embryos die later during development (between 10.5 and 12.5 dpf), and lethality is associated with increased in apoptosis and exencephaly development [133]. Trx2 KD in zebrafish increases apoptosis and induces developmental defects in the liver [134]. In chick, Trx2 KD impairs post-mitotic neurons and induces massive cell death [135]. Thioredoxin reductases (TrxRs) KO reveals that TrxR1 (non-mitochondrial form) is embryonic lethal (E10.5) with multiple abnormalities in all organs except heart [136], whereas TrxR2 KO leads specifically to haematopoietic and heart defects [137]. These works demonstrate a clear dichotomy in the tissue specificity of TrxR1 and 2 actions during development: broad or restricted to heart and haematopoietic lineage development. In addition, Trxs play critical roles in immune response, cancer (for review [138]), and various pathologies in the nervous system (for reviews [72,74]).

#### 4.1.4. Peroxiredoxin Systems in Embryonic Development

The peroxiredoxins (Prxs) family includes 6 members in vertebrates. These enzymes have been mostly studied in physio-pathological contexts or ex vivo where it was demonstrated that Prxs are major regulators of cell adhesion and migration [139]. In vivo, Prxs regulate cadherin expression during early *Drosophila* development [140]. Prxs are typically broadly expressed in embryos with mild specificity amongst isoforms. However, an exhaustive analysis in *Xenopus laevis* reveals maternal expression of prx1, 2, 3 and 6, which persists through all developmental stages. In contrast, Prx4 mRNA becomes detectable at gastrulation and increases afterwards, and Prx5 mRNA is always detected but at low levels. Additional specificities are revealed by in situ hybridization. Specifically, Prx1 is expressed in anterior structures, and Prx4, 5 and 6 expression is preferentially detected in somites [141]. This pattern of expression is not completely conserved between *Xenopus* and other vertebrates. In zebrafish, Prdx1 is expressed in developing vessels, and Prdx1 KD induces vascular defects [97]. In mice, proteomic analysis suggested that prx1 is involved in digit formation where it regulates interdigit apoptosis [142]. One of the best examples of Prxs’ roles during normal differentiation is the involvement of Prx1 and Prx4 in the formation of motor neurons in the spinal cord of chicken and mouse [143,144].

Sexual reproduction and more precisely gamete formation is an unusual process regarding redox signalling. NOXs are involved in spermatogonia and germline stem cell renewal [145]. Moreover, a significant amount of H_2_O_2_ is needed to favour disulfide bridge formation during spermatozoa maturation and capacitation (for review [146]). Conversely, ROS insult should be neutralized to protect DNA and maintain genomic integrity from generation to generation. Several Gpx members are involved in these opposite aspects of redox signalling [147].

### 4.2. Adult Stem Cells and Tissue Homeostasis

During life, adult stem cells replenish damaged and lost tissues. In recent years, H_2_O_2_ appeared to be a major component of stem cell niche, stem cell renewal and recruitment for differentiation (for review [148,149]). In some studies, an increase in H_2_O_2_ is responsible for stem cell differentiation. In *Drosophila*, increased H_2_O_2_ induces the differentiation of haematopoietic progenitor cells, whereas a reduction delays the expression of differentiation markers [150]. In mammals, an increase in H_2_O_2_ induces vascular muscle cell [151] or blood stem cell [152] differentiation. In an apparent contradiction, high H_2_O_2_ levels are associated with stem cell renewal and proliferation. The renewal of intestinal stem cells in *Drosophila* is dependent on H_2_O_2_ [153], and self-renewal of neural stem cells is under the control of NOX activity in the mouse [154,155]. These opposite effects of H_2_O_2_ illustrate our limited understanding of cell fate regulation by redox signalling but both strengthen the relevance of ROS levels in the control of stem cell behaviour and the need for their tight regulation. Some clues come from the induced pluripotent stem cell (iPSC) field where H_2_O_2_ increase is essential during the early phase of iPSC generation. Reduction of H_2_O_2_ levels by NOX KD or antioxidant treatment suppresses nuclear reprogramming [156]. NOX2 is also involved in iPSc differentiation into endothelial cells [157].

### 4.3. Regeneration

**Figure 3:**
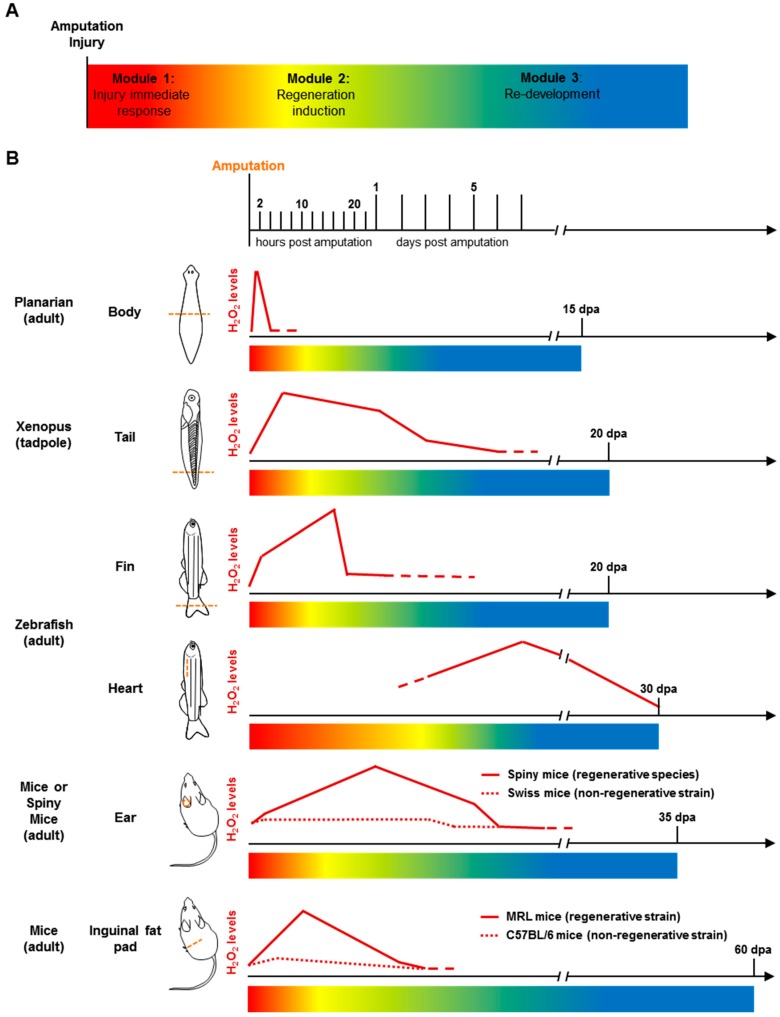
H_2_O_2_ detection during metazoan regeneration. (**A**): regeneration is divided in three modules. (**B**): H_2_O_2_ levels during regeneration in different models and organs. dpa: days post amputation. Adapted from [164,165,166,167,168,169,170].

Some species have the ability to regenerate damaged or removed body parts at adulthood [158,159,160]. Regeneration is probably the best paradigm of adult morphogenesis. The first step of regeneration is wound repair and the formation of a wound epidermis. Shortly after wound epidermis formation, progenitor cells are generated via stem cells that are recruited and the dedifferentiation of differentiated cells [159,161,162]. The newly formed progenitors (and stem cells in some systems) migrate to the wound epithelium to form a mass of undifferentiated cells called the blastema. The entire missing structure will be formed by differentiation and morphogenesis of blastema cells. The process of regeneration can be divided into 3 modules: (1) injury immediate response (wound healing), (2) regeneration induction (blastema formation), (3) formation of the missing structure (blastema growth, differentiation and patterning) [163] (Figure 3A).

It has been known for a long time that wounds generate ROS, specifically H_2_O_2_, in phylogenetically diverse organisms, such as *Drosophila melanogaster*, *C. elegans*, *Danio rerio*, [171,172,173,174], and plants [175,176,177]. In zebrafish, H_2_O_2_ production triggered by wounding is restricted to the first two hours following injury in larva [172] and adult [166]. After a lesion is generated, a gradient of H_2_O_2_ formed by NOX activity is required for leukocyte recruitment to the wound [172,178]. The role of H_2_O_2_ is not restricted to wounding and healing but extends to other steps of regeneration. When a body part is removed, induction of the regenerative programme correlates with a sustained production of H_2_O_2_ for several hours (Figure 3B) [166]. It has been demonstrated in different model organisms that after amputation, H_2_O_2_ signalling not only modulates the regeneration process but is indispensable for launching it (Table 2). Planarian and Hydra regenerate not only body parts but also their body axis [160,179]. In these animals, ROS are detected at the level of the amputation plane shortly after amputation, and reduction of H_2_O_2_ levels impairs regeneration [164,180] (Figure 3B). In *Drosophila*, regeneration can be observed in imaginal discs during larval development and adult gut regeneration [181,182,183]. In both systems, regeneration is redox-dependent [153,181,182,184,185,186,187]. In the *Xenopus* tadpole tail regeneration model, amputation-induced H_2_O_2_ production is necessary to activate Wnt/β-catenin, Fgf signalling and acetylation of lysine 9 of histone 3 (H3K9ac) [165,188] (Figure 3B). In the newt, ROS production is also necessary for neural stem cell proliferation, neurogenesis, and regeneration of dopamine neurons [189]. Moreover, recent data demonstrate that H_2_O_2_ activates voltage-gated Na^+^ channels, linking redox signalling to bioelectric signalling during regeneration [190]. During gecko tail regeneration, H_2_O_2_ is produced by skeletal muscles and is also required for successful tail regeneration. In this case, H_2_O_2_ levels control autophagy in the skeletal muscles and consequently the length of the regenerated tail [191]. In adult zebrafish heart, caudal fin and superficial epithelial cells regeneration models, perturbation of ROS levels through the inhibition of NOX or overexpression of catalase impairs regeneration (Figure 3B) [166,167,192,193]. Different targets were identified at the cellular (neural cells), functional (apoptosis), and molecular levels (MAP kinases and Sonic Hedgehog). In mammals, regeneration at adulthood is very limited [194]. The comparison of regeneration of large circular defects through the ear pinna between regenerative mammals (*Acomys cahirinus*) and non-regenerative mammals (*Mus musculus*) revealed a strong correlation between H_2_O_2_ levels after injury and regenerative capacities (Figure 3B) [168]. In rats, it was further demonstrated that H_2_O_2_ participates in liver regeneration after partial hepatectomy. In this case, sustained and elevated H_2_O_2_ levels activate MAP kinase signalling that triggers the shift from quiescence to proliferation [195]. Recently, it has been demonstrated that H_2_O_2_ produced shortly after inguinal fat pad damage is responsible for its regeneration in MRL mice (Figure 3B). In the non-regenerative C57BL/6 strain, artificial enhancement of H_2_O_2_ leads to regeneration [169].

## 5. Conclusions: Towards the Redox Code

Redox signalling interacts directly or indirectly with most of the signalling pathways that control embryonic development. However, we are only starting to perceive the tip of the iceberg [197]. A comprehensive understanding of developmental redox biology will benefit from a better characterization of thiol targets. For this purpose, the optimization of redox proteomics and the in silico identification of reactive thiols susceptible to redox regulation based on 3D rather than 2D models are two promising strategies [198]. One can note that, unlike other posttranslational modifications (i.e., phosphorylation or ubiquitination), which behave as binary switches, thiol modifications are diverse, including the formation of sulfonic acids (–S–OH), S-nitro groups (S–NO) and disulfides bridges [199]. Each of these modifications could confer a specific status to the targeted protein, thus extending the spectrum of regulation provided by redox signalling [200]. Finally, because the different members of the redox machinery are interconnected, the modification of a specific thiol likely depends on the equilibrium of the entire redox machine. Modelling the entire process by integrating dynamic and quantitative information of the different redox machinery members would greatly help to decipher the physiological role of redox signalling.

## Figures and Tables

**Table 1:** Expression of the redox machinery genes during *Danio rerio* development.

Enzyme	Gene	Cellular Localization	Gene Expression	Reference
**Catalase**	cat	mitochondria, peroxisome	brain, digestive system, gill, muscle, sensory system	[86]
**Glutaredoxin**	glrx2	cytoplasm	whole organism	[87]
	glrx3	cytoplasm	brain, heart, sensory system	[86,88]
	glrx5	cytoplasm	blood island, digestive system, heart, sensory system	[89]
**Glutathione Peroxidase**	gpx1a	nd	digestive system, muscle, sensory system	[90,91]
	gpx1b	cytoplasm	digestive system, sensory system	[86,92]
	gpx4a	nd	digestive system, peridermis	[90,93]
	gpx4b	nd	blastoderm, digestive system, epidermis, epiphysis, muscle, pharyngeal arch, pronephric duct, sensory system	[90,93,94]
	gpx7	nd	notochord, splanchnocranium	[86]
	gpx8	membrane	notochord, pharyngeal arch, sensory system	[86]
**Glutathione Reductase**	gsr	cytoplasm	digestive system, macrophage	[86]
**NADPH Oxidase**	nox1	membrane	brain, spinal cord, sensory system	[79]
	nox2/Cybb	membrane	blood, brain, spinal cord, sensory system	[79,86]
	nox5	membrane	brain, spinal cord, sensory system	[79]
	duox	membrane	brain, digestive system, epidermis, spinal cord, sensory system, swim bladder, thyroid,	[79,95,96]
**Peroxiredoxin**	prdx1	cytoplasm	brain, neural crest derivatives, vessels	[97,98]
	prdx2	nd	blood, CNS, digestive system, pharyngeal arch, sensory system	[90]
	prdx3	nd	blood, digestive system, myotome, pharyngeal arch, sensory system	[90]
	prdx4	nd	digestive system, hatching gland, pharyngeal arch, sensory system	[86,99]
	prdx5	nd	macrophage, pronephric duct, sensory system	[86]
	prdx6	nd	digestive system, rhombomere, sensory system	[86]
**Superoxide Dismutase**	sod1	cytoplasm	whole organism	[86,100]
	sod2	mitochondria	blood, brain, digestive system, gill, kidney, muscle, sensory system	[90,101]
	sod3b	cytoplasm	whole organism	[102]
**Thioredoxin**	txn	nd	digestive system, Hypophysis, spinal cord, sensory system, tegmentum	[86,91]
	txn2	mitochondria	whole organism	[86]
**Thioredoxin Reductase**	txnrd3	mitochondria	blood, CNS, digestive system, muscle, pharyngeal arch, spinal cord, sensory system	[86,90]

No results have been reported for gpx2, gpx3, gpx9, nox4, sod3a, txnrd2-1, and txnrd2-2. CNS: central nervous system.

**Table 2:** Redox regulation of regeneration among Phyla. APO. apocynin; DHE: dihydroethidium; dpa: days post-amputation; hpa: hours post amputation; n.s.: no significant.

Classification	Animals/Species	Stage	ROS Detection	Profil	ROS Modulation	Organ/Appendage	ROS Targets	Reference
Cnidaria	Hydra		DMPO	wound edge				[180]
Platyhelminthes	Planarian (*Schmidtea mediterranea*)		H_2_DCFDA	burst at the wound site	DPI, APO	central nervous system	neuroregeneration	[164]
Arthropoda	Drosophila (*Drosophila melanogaster*)	Larvae	CellRox green	burst after apoptosis induction	NAC, vitamin C, Trolox, SOD, CAT	wing imaginal disc	p38 pathway	[181]
							JNK pathway	
			DHE, H_2_DCFDA	up to 24 h after apoptosis induction	misexpression of extracell.CAT	eye and wing imaginal disc	macrophages	[182]
		Adult	H_2_DCFDA	burst after oral admin. of HgCl2	Vitamin E	midgut		[183]
Amphibians	Xenopus (*Xenopus laevis or tropicalis*)	Tadpole	HyPer	production 6 h-4 dpa	DPI, APO, MCI-186	Tail	Wnt/b-catenin pathway	[165]
							FGF pathway	
			DHE	nd	DPI, MCI, VAS, H_2_O_2_		bioelectric activity	[190]
			H_2_DCFDA	nd	APO	Notochord in tail	acetylation of H3K9	[188]
Squamata	Gecko (*Gekko japonicus*)	Adult	H_2_DCFDA	production (0-7 dpa), skeletal muscles	DPI, APO	tail	autophagy in skeletal muscles (ULK, MAPK)	[191]
Teleost fish	Zebrafish (*Danio rerio*)	Larvae	HyPer	nd	DPI	caudal fin	Src family kinase	[196]
			PFBS-F				*ptch1, tcf7, raldh2, pea3, ihhb*	[91]
		Adult	H_2_DCFDA	production 0-16 hpa	VAS2870, DPI		Apoptosis	[166]
							JNK pathway	
			H_2_DCFDA, HyPer		VAS2870, H_2_O_2_		Hh pathway	[193]
							nerve	
			Myl7:HyPer, Redox sensor cc-1	production 3-14 dpa epicardium and adjacent myocardium, max 7 dpa	DPI, APO, CAT	heart	ERK pathway	[167]
			CellRox green	production 2 h-12 hpa	VAS2870	superficial epithelial cells (SECs) in caudal fin		[192]
Mammals	Rat (*Rattus norvegicus*)	Adult	H_2_DCFDA, red H_2_O_2_ assay kit, amplex red H_2_O_2_ assay kit	production 1 h-3 dpa	GOX, CAT	liver	ERK pathway	[195]
							p38 pathway	
	Mice (*Mus musculus*)	Adult	luminol	MRL mice (production 0-72 h pa, max 12 h), C57Bl6 (n.s. production)		inguinal fat pad		[169]
			lucigenin	n.s. production 3 h-10 dpa		ear		[168]
			luminol	production 3h-4 dpa				
	Spiny mice (*Acomys cahirinus*)	Adult	lucigenin	production 3 h-5 dpa				
			luminol	n.s. production 3h-10 dpa				
